# Altruism the Essense of the Iranian Nurses’ Job Satisfaction: A Qualitative Study

**DOI:** 10.5539/gjhs.v8n8p13

**Published:** 2015-12-17

**Authors:** Alireza Nikbakht Nasrabadi, Zahra Sadat Dibaji Forooshani, Forough Rafiee

**Affiliations:** 1 Department of Nursing, Faculty of Nursing and Midwifery, Tehran University of Medical Science, Tehran, Iran; 2 Department of Nursing, School of Nursing and Midwifery, Tehran University of Medical Sciences, International Campus (TUMS-IC), Tehran, Iran; 3 Department of Nursing, School of Nursing and Midwifery, Iran University of Medical Sciences, Tehran, Iran

**Keywords:** altruism, job satisfaction, qualitative research, Iran

## Abstract

Skillful and efficient human resource is one of the most important tools for reaching the organizational targets and it is almost impossible to reach the predetermined goals and success without having skillful human resources. Therefore, having a study on the personnel’s job satisfaction is recommended for all of the organizations. Since the health organizations are among the most important organizations of any country, paying attention to the nurses’ job satisfaction as the main providers of the health care services gets very important. In fact, their attempts guarantee the efficient human resources’ health in the society. Understanding the Iranian nurses’ experiences of their job satisfaction. The present paper studies the implicit and explicit aspects of the clinical nurses’ job satisfaction. The needed information is collected via interviews, and then the participants’ contextual data is analyzed by the qualitative content analysis. The research results introduce the altruism as the foundation for the nurses’ job satisfaction. Altruism is composed of three categories of the patient advocacy, spiritual job satisfaction, and professional commitment. Altruism has made the nurses deliver the required health cares to the patients with all their love, while their profession has many difficulties. Job satisfaction resulted from altruism is experienced as a pleasant feeling along with enjoyment resulted from addressing the needs of a patient who looks forward to the nurse’s advocacy. According to this kind of job satisfaction, the nurse’s professional commitment is to advocate for the patient. Also, the research results show that spirituality is the inseparable component of altruism and it has a vital role in the nurses’ job satisfaction. The spirituality helps the nurses to deliver targeted acts and interventions.

## 1. Introduction

### 1.1 Background

Certainly, skillful and efficient human resource is one of the most important tools for reaching the organizational targets and it is almost impossible to reach the predetermined goals and success without having skillful human resources (Moradi, 2011). Today, the dynamic economic environment and the increase of intention of competition in the field of delivering better services and improving efficiency have made the organizations improve their capability and flexibility. Therefore, the managers have noticed that employees with high motivation and job satisfaction have a considerable effect on determination of job quality within the organization. As a result, the highly motivated and satisfied employees are the most important assets of the organizations (Singh, 2009). Consequently, the organizations try to improve the employees’ job satisfaction in order to cause the organizational motivation and commitment, and also to increase their competitiveness with other organizations. Therefore, having a study on the personnel’s job satisfaction has a special importance in all of the organizations (Arabi & Hutton, 2012).

Job satisfaction has constantly been considered as a noticeable issue in the organizations (Roman, 2008). Since the health organizations are among the most important organizations of any country, they pay too much attention to the human resources. In fact, the attempts of the health organizations guarantee the efficient people’s health. Therefore, paying attention to the inter-organizational satisfaction whether directly or indirectly leads to the improvement of health and services within a society. Since nurses make the main part of the health organizations, -nurses make 80 percent of the personnel in some of the health organizations-nurses’ job satisfaction is highly important. Though the studies on the nurses’ job satisfaction has a far history, the nurses’ needs are usually ignored in the majority of the health cares all over the world. As a result, it leads to the nurses’ turnover, and consequently the nursing shortage appears in most of the world hospitals (Masroor & Fakir, 2010). Therefore, job satisfaction has a considerable importance in nursing.

Ignoring the job satisfaction leads to serious consequences. Gradually, it results in the loss of sense of responsibility and the turnover. However, the nurses will deliver better services if they have a higher job satisfaction regarding mental and physical aspects (Tajvar & Arab, 2006). According to the statistics released, nursing shortage is visible in the world. Australia annually needs 13500 nurses for 10 years. Also it is predicted that Canada would need 113000 nurses up to 2016, moreover, it has been estimated that there would be a 20 percent nursing shortage in America (approximately 581500 nurses) up to 2020 (Rebecca, 2011). It has also been predicted that the constant nursing shortage will be felt in the future (Dimeglio et al., 2005).

The studies indicates that even in the clinical environments of Iran, the nurses are unwilling to deliver health care, they have second jobs unrelated to nursing, they are willing to leave job, they are unsatisfied with the administrative rules, they frequently take time off, and they complain about excessive workload, therefore, the nursing shortage is felt (Soodi & Mortazavi, 2013). According to the research studies, nurses with higher job satisfaction have more efficiency and they do not like to leave their job (MacKusick, 2010). In fact, effects resulted from job satisfaction are widespread and multilateral and they could influence the nurse’s life and job quality (Rebecca, 2011), reduction of job burnout (Dimeglio, 2005), and reduction of job stress (Mihyun, 2012). On the other hand, the improvement of job satisfaction could also improve the nursing cares quality (Rebecca, 2011) and it leads to the patients’ more satisfaction (Streubert & Carpenter, 2003).

Though there are various studies on different aspects of job satisfaction, job satisfaction has a multilateral essence and it depends on the environmental conditions (19.8), therefore, the present paper intends to understand the Iranian nurses’ experiences in the field of job satisfaction.

### 1.2 Objectives

Understanding the Iranian nurses’ experiences of their job satisfaction.

## 2. Materials/Patients and Methods

The present paper is part of a comprehensive study related to explaining the nurses’ job satisfaction process based on their experiences. The study intends to identify and explain the nurses’ understanding of their job satisfaction. Since the aspects of job satisfaction are influenced by the organizational culture and the nurses’ beliefs and attitudes, the research is done based on the qualitative method. The qualitative method is more appropriate for obtaining rich data and clarifying the social processes inherent in the human interactions (Tovey & Adams, 1999). The sample size in the study is selected purposefully. The sample size consists of 10 nurses (4 male and 6 female) with the job experience varying from 1 year to 25 years. All of the participants have the B.A. and as the sector employees they take direct care of the patients. They work in the public and special sectors of the governmental and non-governmental hospitals located in Tehran.

In the study, the semi-structured interview is used as the data gathering method. Before doing the interview, the participants have got informed of the research purpose and they have delivered their written satisfaction for taking part in the study. Moreover, their interviews have been recorded after receiving their permission. The interviewed individuals have got assured that their personal information would not be revealed in the published reports; moreover, they could have access to the research results.

The interview includes 18 sessions and the interview duration varies from 50 to 70 minutes depending on the participants’ responding. After the interview, it has been written word by word, and then it has been analyzed by the qualitative content analysis.

The qualitative content analysis is an analytical method for mentally interpreting the content of the contextual data (Hsieh & Shannon, 2005). According to the mentioned method, the codes and classes are directly and inductively extracted from the raw data through the systematic classification process (Zhang & Wildemuth, 2008). The content analysis is beyond the extraction of the explicit content taken from the contextual data. In this way, it is possible to reveal the key concepts and implicit patterns inherent in the participants’ data content (21). In the process of qualitative content analysis, the data gathering and analysis are done simultaneously (Zhang & Wildemuth, 2008). Moreover, the analytical units are selected from the interview text. The analysis unit is the analytical part of the text that helps to reach the research purposes. The primary codes of the meaning units which are the important part of the analysis unit will be extracted. The primary codes could include the real content of the participants’ interview or the researcher’s understanding and analysis of the content. Then, the primary codes are classified into subclasses based on the differences and similarities. Finally, the subclasses are divided into the abstract classes and the key concepts (Zhang & Wildemuth, 2008).

In order to determine the validity, the research has applied the information retrieval done by the participants and the data study done by the research team. For the information retrieval, the participants’ speech and experiences were given back to them, moreover, the researcher gave them back a complete typed text of the first four interviews along with the primary codes in order to be confirmed or edited. However, all of them were confirmed. Moreover, the encoding and the primary classes were received by the research team members in order to be analyzing data and confirmed. In order to increase reliability, the researcher has a more constant relation with the participants and also he/she has dedicated enough time to the interviews. Moreover, the researcher has provided the proper and friendly environment for the participants in order to obtain their trust, and consequently reach their more accurate experiences. The participants were completely willing to take part in the interview and to express their feelings, experiences, and thoughts. The researcher has also tried to write his own thoughts and to keep his thoughts far from the research so that they could not appear in the research which should include the participants’ experiences.

## 3. Results

The results of the data analysis show that the majority of the participants believe that the altruism is the foundation for their job satisfaction. According to the qualitative content analysis resulted from the participants’ interview, the altruism is the main essence of the nurses’ job satisfaction and it is composed of three subcategories of the patient advocacy, spiritual job satisfaction, and the professional commitment. The three subcategories are described as follows:

Patient advocacy. Many of the participants in the study have mentioned that all of the patients need help and it affects the participants’ job satisfaction to advocate for the patients. One of the nurses expresses his feeling” it does not matter whether the patient is highly literate or completely illiterate, because all of them need help and it is very important for me to be able to advocate for all of them”.

Another nurse describes the patient advocacy in this way, “the patients or their families have hope in what the medical team especially the nurses do, because the nurses are at hand and they ask for their advocacy even in relation to the smallest problems”. An experienced nurse describes the patient advocacy and says” there is a sense of satisfaction in nursing. When you see that people need help and you can help them, then you can sleep with an easy conscience”. Another nurse describes the nursing difficulties and says” there are many difficulties in nursing. A nurse has many duties, he has job difficulties, and he has to take shifts, however, when you see that the patients ask you for help with all their hearts, you would feel that you are useful for them”. One of the other nurses who have a working experience of 13 years describes his feeling and says “it is true that we ignore ourselves during the night shifts, however, what matters is that we are useful especially when the patients need us”.

Another participant declares that sense of being useful and supporting the patient are crucial for a nurse not to leave his job. He says” in my opinion, job satisfaction means having the feeling of being able to help a person who needs help. The help might be very small such as the patient’s need for speaking with someone or it might be grave such as the patient’s need for advocacy and it could be understood from the patient’s words or behaviors. Anyhow, I really enjoy my job”.

One of the male nurses describes his satisfaction and says” the men enjoy supporting those who need help and this issue is true even in their personal lives. Therefore, it makes us happy to advocate for the patients or their families who ask for help”. Another nurse says” when you are really sad with your boss or coworker, you will forget your sadness by seeing a person’s or a patient’s need for help and you will do your best to help him”. The patient advocacy could be revealed in different ways. One of the nurses say” sometimes that patients have a fight with the nurses; I feel that they do not mean to fight but to draw the nurses’ attention to themselves and have their advocacy. In fact, they ask for help and a sense of satisfaction is obtained when we help them”.

### 3.1 Spiritual Job Satisfaction

Majority of the participants of the study introduce nursing as a spiritual and holly job. They believe that all of the people who select nursing as their job know that the spiritual satisfaction inherent in doing the duties is a very important aspect of their job satisfaction. They declare that the nurses’ attempts to meet the patients’ needs are very important to reach the spiritual job satisfaction, and finally the job satisfaction itself. In this relation, one of the nurses say” when I face the patients, I put myself in their shoes and I do my best to have a behavior with them that a mother might have with his sick child. Even if they do not respect me, I will do my best to keep calm. The feeling of helping someone makes me calm and I feel that it is a kind of praying, I even feel the effect of nursing on my life. When I see the nursing from this point of view, I really find it beautiful”.

Another nurse says” it is true that the financial needs matter; however, our job is holly. During my working experience, I have had different patients some of whom have died, but I have also had patients, whom kept me awake all night long, and then they survived and it has brought me sense of satisfaction”.

Another nurse says” I enjoy helping patients. It increases the spirituality of my life”. In relation to the spiritual job satisfaction, a nurse says “During my working years, when a patient is satisfied and gets out of the hospital with satisfaction he leaves me a blessing and it helps me not pay too much attention to my problems. I would be calm and leave my workplace with a sense of satisfaction”. Another nurse with a working experience of 20 years says” the most important delight for me is the patients’ blessing for me”. The belief in the spiritual satisfaction for some of the participants is so effective that they find the nursing problems more bearable. A nurse says” we have no one to support us and our soul and body are hurt, however, when we feel that someone prays for us it gives us the hope that someday and somehow his blessing would help us. Therefore, we bear the difficulties of nursing such as sleeplessness, insults, low salaries, and so forth…”

### 4. Professional Commitment

In this research, the responsibly taking care of the patients without considering the conditions and the working problems is one of the important factors that affect the nurses’ job satisfaction. In this condition, the patients pay attention to the correctly performing of their duties without considering the results or rewards. In this relation, a nurse says” when I do my job, even if it has no good results-for instance when I do the CPR-, and even if my patient dies, I will have a sense of satisfaction because I know that I have done my best and I could do no more.”

Another nurse describes his feeling in this way” it does not matter what happens at the end. What satisfies me is that I do my best and I do my duty professionally to help a human, then I have an easy conscience. This feeling only belongs to the jobs that deal with the living things, while in other professions what matters is the result not the process”. One of the participants of the present paper says” nursing is a difficult job, it has many responsibilities, but what matters is that I can do my duty correctly and it satisfies me. This feeling is very strong in nursing”.

In relation to the professional commitment related to the job satisfaction, another nurse says” if I do my job correctly and based on standards, I will be happy with my job and I will experience a good feeling related to doing my best for the patients. Even if my position is ignored professionally, I will have sense of satisfaction”. Another participant says”, if I do not do my job accurately, I will not have an easy conscience at home and I will tell myself that my patient could have reached health sooner, if I had done my job properly”.

## 5. Discussion

As it has been discussed, altruism is one of the important components of job satisfaction in the present paper and it refers to helping others (Soosai-Nathanl et al., 2013). In fact, altruism means helping others without the direct or indirect expectation for compensation (Wendy, 2011) and it leads to the individuals’ sense of being well and healthy, and also sense of enjoyment resulted from work (Soosai-Nathanl et al., 2013). It also has a role in motivating the nurses.

The present paper shows that one of the crucial reasons that leads to the nurses’ retention is the altruism. Altruism is composed of three categories of patient advocacy, spiritual job satisfaction, and professional commitment. According to the study, majority of the nurses believe that their job satisfaction results from helping the patients. The necessity of patient advocacy has roots in the patients’ independence and decision making capability and it gives the nurse a dominant position. Mostly, in the health-care environments, the patients feel powerless and weak; therefore, they are seriously vulnerable. As a result, the importance of the nurses’ role as the advocates for the patient is highly emphasized (Negarandeh et al., 2000). Therefore, the international council of nurses included the patient advocacy in its instructions in 1970 (Mallik & McHale, 2004).

The authors who are also nurses have different views on what is called” advocacy” in nursing. According to the legal and moral frameworks, the advocacy is the philosophy of nursing and it includes special acts such as helping the patient to have access to the required health-cares, guaranteeing the quality of health-care, defending the patient’s rights, and making a connection between the patient and the health-care systems (Mahmoudi, 2013).

Leddy et al. believe that advocacy in nursing refers to informing and supporting the patient so that he could make the best possible decision for himself. Therefore, advocacy guarantees the patient’s rights and values and also respecting individuals (Leddy & Pepper, 1993).

Also, in relation to the patient advocacy, Ingram believes that advocacy is the innate element of the nursing ethics; moreover, the moral behavior and advocacy are the nurse’s duty (Ingram, 2003).

In relation to the second finding of the study-spiritual job satisfaction-the participants believe that the patient’s praying for them causes blessing in their lives and meeting the patient’s ‘needs brings peace to their personal lives. Moreover, the nurses have experienced the spiritual job satisfaction as a pleasant feeling along with enjoyment which results from meeting a patient’s needs. The nurses believe that in Islam taking care of patients is based on the divine inspirations (Ravari et al., 2009).

The results of the study indicate that spirituality is an inseparable component of nursing, and also it is an inherent aspect that could courage the nurses delivers targeted acts and interventions. According to Florence Nightingale, a good nurse is a person who could feel God in his work.

One of the other research finding refers to the professional commitment. Regarding professional commitment, taking care of the patient finds a different definition. The nurses declare that nursing correctly and professionally without considering the obtained results could cause sense of satisfaction. According to the growing and dynamic system, nursing needs nurses who deliver desired and correct health-care services. Therefore, the nurses need to have a proper functional performance, high sense of judgment and critical thinking, clinical decision making ability, ethical concluding, and effective relation with the patient (Ravari et al., 2009).

The everyday development of the care-based-sciences has caused complicated problems in the nurses’ function in the clinical environments. The problems could make difficulties for recognizing and correct responding abilities in taking care of the patients (Mallik & McHale, 2004). Disregard of the potential risks that might threaten the nurses, the nurses need a constant commitment to the ethics in order to do their job correctly (Rafati & Rezheh, 2012).

Regarding the professional commitment, the commitment to the patients is more important than the concerns threatening nurses in relation to the possible risks. The nurses could fade their fears and they know how to give the best services to the patients according to the existing conditions (Mohammadi et al., 2013). In such conditions, the nurses do not care about the consequences and they only attempt to do their duties correctly and professionally in order to reach the final goal (Mohammadi et al., 2013).

Generally, majority of the participants in the study combine the valuable concepts such as supporting the patient who needs help, having sympathy with the patient, having sense of responsibility for the patients, and doing the nursing tasks professionally in order to help the patients.

A review of the studies shows that in relation to the professional commitment and patient advocacy, no independent research has been done. However, according to the research results related to the spiritual job satisfaction it is clarified that the results of the present paper are in agreement with the results of a study done by Ravari et al. (2011) titled as the spiritual approach to the nurses’ job satisfaction. The results of the present paper also shows that spirituality is an inseparable component of nursing and it is also an inherent aspect of nursing that could courage the nurses deliver the targeted acts and interventions, moreover, they will be able to have an effective and friendly relation with God and patients. Spiritual job satisfaction could have a considerable role in bearing the difficulties of nursing, and also it could make the nursing acts enjoyable. Moreover, the results of the present paper are in agreement with the results of a study done by Emami et al. (2007). Emami et al., considered three groups of the Iranian nurses working in Iran and abroad as the sample of the study. The results of their study showed that the moral values and ethical issues were one of the most important aspects of the nurses ’job satisfaction.

Conceptually, the concept of job satisfaction is a single concept that could not be analyzed; therefore, different people might define it ambiguously. Then, job satisfaction could both be considered as a feeling or it could refer to a person’s attitude toward part of his job (Ingram, 1998). It reveals that job satisfaction is a single concept; however, it could have various aspects. In this study, nurses mentioned their dissatisfaction with the nursing problems; however, they declared that they did not leave their job because of the positive feeling experienced as a result of helping the patients. Consequently, this aspect of job satisfaction should highly be enhanced.
